# Invasive observation of reactive systolic blood pressure responses to upper-arm cuff inflation

**DOI:** 10.1038/s41371-026-01172-3

**Published:** 2026-06-17

**Authors:** Louisa F. Morris, Matthew K. Armstrong, James E. Sharman, Martin G. Schultz, J. Andrew Black, Nathan B. Dwyer, Philip Roberts-Thomson, Heath Adams, Stéphane G. Carlier, Dean S. Picone

**Affiliations:** 1 Menzies Institute for Medical Research, University of Tasmania, Hobart, Australia; 2 Department of Exercise Science, Falk College of Sport, Syracuse University, Syracuse, USA; 3 Department of Rural Health, University of Melbourne, Shepparton, Australia; 4 Royal Hobart Hospital, Hobart, Australia; 5 University of Mons (UMons), Faculty of Medicine, Department of Cardiology, Mons, Belgium; 6 School of Health Sciences, Faculty of Medicine and Health, The University of Sydney, Sydney, Australia; 7 Westmead Applied Research Centre, The University of Sydney, Sydney, Australia

**Keywords:** Diagnosis, Medical research

## Abstract

Non-invasive blood pressure (BP) measurement is performed by the inflation of an upper-arm cuff, which itself could induce a reactive BP response and potentially influence BP management. The aim of this study was to determine if cuff inflation was associated with reactive BP responses. Beat-to-beat invasive aortic BP was measured continuously before and during inflation of an automated upper-arm cuff in 234 participants (61 ± 10 years, 31% female) undergoing coronary angiography. Reactive responses were calculated as invasive aortic systolic BP immediately post cuff inflation minus baseline aortic systolic BP immediately pre-cuff inflation. Three groups were defined based on an increase (≥ 5 mmHg), decrease (≤ −5 mmHg) or no response (>−5–<5 mmHg) in aortic systolic BP. Cuff inflation was associated with an average increase in aortic systolic BP of 9.8 ± 5.1 mmHg in 28 (12%) participants, an average decrease of 9.2 ± 5.4 mmHg in 43 (18%) participants, and no response in 163 (70%) participants (−0.3 ± 2.4 mmHg). Cuff and baseline aortic systolic BP were higher in participants who exhibited a reactive response and BP variability was greater among the decrease group (ANOVA p < 0.05). No clinical characteristics were associated with reactive responses. Similar findings were observed for diastolic BP, mean arterial pressure and pulse pressure. Approximately one-third of participants exhibited a substantial reactive systolic BP response (increase or decrease) to upper-arm cuff inflation as determined from invasive BP measurements. Whether this could influence the accuracy of cuff BP measures, and consequent hypertension management, should be determined in future, prospectively designed studies.

**Figure:**
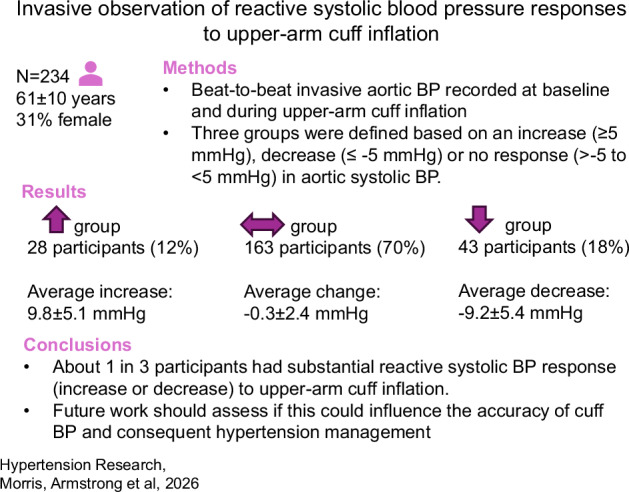

## Introduction

Cardiovascular disease (CVD) stands as the foremost cause of morbidity and mortality globally [1]. High blood pressure (BP) is the most significant modifiable risk factor for CVD, and effectively managing high BP substantially reduces the incidence of CVD morbidity and mortality [2]. Given these factors, accurate BP measurement is essential to guide diagnosis and management of high BP [3]. The process of measuring BP, which traditionally involves inflating a cuff around the upper arm, may itself trigger a response in BP which could result in an acute change in BP [4, 5]. This immediate BP change is hypothesised to result from the alerting effect of cuff inflation, or the anxiety associated with the BP measurement.

If the BP response triggered by cuff inflation is substantial enough to change the classification of hypertension, it could impair the accurate diagnosis and management of high BP. To date, investigations into the reactive BP response to cuff inflation that have used invasive BP as the reference measurement have varied in terms of the interval between cuff inflations, the number of cuff inflations, the peak cuff inflation pressure, the location of invasive BP measurement, and the analysis method. The acute reactive change in invasive BP is most often an increase [4, 5], although one study has demonstrated a small proportion of people may also have a decrease in BP [4] and another observed no effect of cuff inflation on invasive BP [6]. Further, one study has used invasive central aortic BP monitoring to test reactive responses to cuff inflation however, the cuff was both inflated and deflated at a rate of 20 mmHg/s which is not in accordance with standard cuff BP measurement methods [5]. Therefore, the primary aim of this research was to determine if cuff inflation is associated with a reactive BP response confirmed with invasive aortic BP monitoring. Furthermore, this investigation examined the (theoretical) possibility that such a response to cuff inflation might influence the classification of hypertension. An additional goal included exploring if there were demographic and clinical characteristics associated with reactive BP responses.

## Methods

### Study overview

The study population comprised individuals scheduled to undergo elective coronary angiography at the Royal Hobart Hospital, Australia. All individuals arrived at the Royal Hobart Hospital after abstaining from coffee, alcohol, and food overnight or for at least eight hours prior to study procedures. A total of 390 individuals were approached for study participation, and six declined to participate. Participants were excluded from the study on the basis of: atrial fibrillation (n = 15), aortic stenosis (n = 5), age >80 years old (n = 2), inter-arm BP difference >5 mmHg (n = 17), technical or equipment error during data collection (n = 59), medical complications during the angiogram (n = 17), femoral artery access (n = 21), haemodynamic irregularities such as ectopic beats (n = 12) or if the baseline invasive BP measurements were not available (n = 2). After exclusions, data were available for 234 participants. All study participants provided written informed consent and ethical approval was granted by the Health and Medical Human Research Ethics Committee of Tasmania (H0016939) and the study was conducted in accordance with the Declaration of Helsinki. The protocol was designed to compare cuff measured BP with invasive BP which has been previously reported [7, 8], and this is an hypothesis generating, secondary analysis of the data. The data and statistical code will be made available to other researchers upon reasonable request to the corresponding author.

### Automated cuff BP inflation and measurement

Prior to commencement of the coronary angiography procedure, all participants were fitted with an appropriately sized upper-arm cuff. The upper-arm cuff was connected to an automated oscillometric device which was used for all non-invasive BP measurements during the coronary angiography procedure (SphygmoCor Xcel, AtCor Medical, Sydney Australia). The cuff inflated to a maximum pressure that was automatically determined by the device (which usually approximates 30 mmHg above systolic BP for this type of device) before steady deflation to measure BP via the oscillometric method. Each participant was informed that the research protocol would include cuff BP measurement and were asked to remain quiet and still while the measurements were recorded. There was no standardized warning given to participants as to exactly when the cuff would start inflating. Only the first of two cuff measurements was used for the analysis, to ensure the reactive response that was observed was not influenced by prior cuff inflations.

### Invasive BP

Invasive BP data were recorded using an analogue to digital converter at a frequency of 1000 Hz (PowerLab AD Instruments Bella Vista, New South Wales, Australia) and collected according to consensus guidelines [9]. A fluid-filled catheter (e.g. 5 F TIG, 5–6 F Judkins Left, 5–6 F multipurpose) was positioned in the ascending aorta and continuous invasive BP waveforms were recorded immediately prior to cuff inflation, referred to herein as ‘baseline measurement’. Following capture of the baseline measurement, the upper-arm cuff was inflated and invasive BP waveform recordings from the first 10 s following the start of cuff inflation were recorded and used for analysis (herein referred to as ‘cuff inflation measurement’). Simultaneous to the onset of cuff inflation, a digital marker was added to the invasive BP recording (Labchart 7, AD Instruments Bella Vista, New South Wales, Australia). The catheter was flushed prior to waveform recordings. Invasive systolic and diastolic BP, pulse pressure, mean arterial pressure, and heart rate were calculated from continuous invasive BP waveform recordings using a customised script written in R (R Foundation for Statistical Programming, Vienna, Austria) [10].

### Reactive BP response

The invasive BP response associated with cuff inflation was analysed in two five-second periods (period 1 and 2). The reactive systolic BP response as a continuous variable was calculated from the mean systolic BP over the entire baseline measurement period and the mean systolic BP over periods 1 and 2 of the cuff inflation measurement, using the equation:

$${Reactive}\,{systolic}\,{BP}\,{response}=\left({Period}\,1-{Baseline}\,\right)+({Period}\,2-{Period}\,1)$$

A reactive systolic BP response was defined as a ≥ 5 mmHg increase or decrease in systolic BP from baseline to cuff inflation. A threshold of ≥5 mmHg was chosen as it is generally accepted as the upper limit of measurement variability and has previously been used as the threshold to define a reactive systolic BP response [11, 12]. The same calculations were used for diastolic BP, PP, MAP and heart rate. Separately, to include a surrogate of autonomic nervous system activity, systolic and diastolic BP variability were calculated for the baseline and cuff inflation periods as 1) beat-to-beat standard deviation, and; 2) coefficient of variation = (standard deviation of BP ÷ mean BP) × 100.

### Clinical characteristics

Clinical characteristics including medical history and anthropometry were retrieved from hospital digital medical records.

### Theoretical classification of high systolic BP

A theoretical misclassification of high systolic BP was defined as a change in invasive aortic systolic BP from baseline measurement to cuff inflation measurement which crossed the threshold of 130 mmHg, as per the American College of Cardiology/American Heart Association (ACC/AHA) definition of systolic hypertension [13].

### Statistical analysis

Variables were reported as mean ± SD for normally distributed data or median [interquartile range] for non-normally distributed data. Differences between variables were assessed using one-way analysis of variance (ANOVA; continuous variables) with Tukey Honest Significant Difference Tests for post hoc comparisons. Pearson’s Chi-squared tests were performed to compare categorical variables between groups. Multivariable regression was used to assess whether significant bivariate associations with a reactive systolic BP response remained significant after adjusting for potential confounders, such as standard CVD risk factors. Residual versus fitted plots were visually inspected for non-linear or heteroscedasticity. Scale location plots were analysed for homogeneity of residuals. The influence of a short baseline measurement (< 5 s) and medication use on reactive systolic BP response was also assessed in a separate sensitivity analysis. A p value < 0.05 was considered statistically significant.

## Results

### Participant characteristics

Participant characteristics are presented overall, and according to three patterns of reactive systolic BP response (Table 1). Overall, participants were predominantly male, middle-to-older age, and overweight according to body mass index. Most participants had high BP, a family history of CVD and stenosis of at least one coronary artery. Conscious sedation was administered prior to the procedure in most participants (fentanyl: n = 205, 92% and midazolam: n = 198, 89%).

**Table 1:** Clinical characteristics of participants across the three groups in response to cuff inflation.

Variable	All (*n* = *234*)	‘No response’ (*n* = *163)*	‘Decrease’ *(n* = *43)*	‘Increase’ (*n* = *28)*	p value (ANOVA)
Age (years)	61 ± 10	60 ± 10	62 ± 10	63 ± 11.0	0.28
Female sex, n (%)	70 (29.9) *NA* = *7*	51 (32.5) *NA* = *6*	13 (31.0) *NA* = *1*	6 (21.4)	0.51
Body mass index (kg/m ^2^)	29.6 ± 5.4	29.4 ± 5.4	30.2 ± 5.7	29.8 ± 5.0	0.72
Positive smoking history, n (%)	102 (48.6) *NA* = *24*	73 (49.7) *NA* = *16*	14 (37.8) *NA* = *6*	15 (57.7) *NA* = *2*	0.27
High BP, n, (%)	155 (66.2) *NA* = *18*	104 (69.3) *NA* = *13*	31 (79.5) *NA* = *4*	20 (74.1) *NA* = *1*	0.44
Family history of CVD, n (%)	134 (57.3) *NA* = *26*	99 (66.9) *NA* = *15*	25 (71.4) *NA* = *8*	10 (40.0) *NA* = *3*	0.021
Type II diabetes mellitus, n, (%)	58 (27.0) *NA* = *19*	41 (27.2) *NA* = *12*	8 (22.9) *NA* = *5*	9 (34.6) *NA* = *2*	0.67
Coronary artery disease, n (%)	162 (69.2) *NA* = *7*	109 (69.4) *NA* = *6*	34 (81.0) *NA* = *1*	19 (67.9)	0.31

Data is expressed as mean ± SD or n (%).

*BP* blood pressure, *CVD* cardiovascular disease, *NA* data not available.

### Reactive response of systolic BP to cuff inflation

**Fig. 1:**
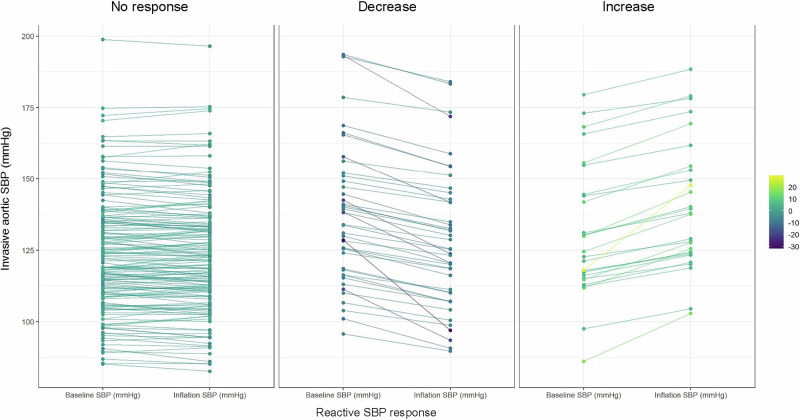
The reactive response in systolic blood pressure (SBP) stratified into no response, decrease, and increase groups. Participants in the decrease group had a higher baseline invasive aortic SBP (138 ± 25 mmHg, p < 0.001) than participants in the no response and increase groups. Participants in the increase group had a higher invasive aortic SBP following cuff inflation (139 ± 23 mmHg, p < 0.001) than participants in the no response and decrease groups.

Three patterns of reactive systolic BP response associated with cuff inflation were observed (Table 2). An increase in systolic BP of at least 5 mmHg was observed among 28 participants (12%), a decrease in systolic BP of at least 5 mmHg was observed among 43 participants (18%) and there was no response (less than 5 mmHg) in 163 participants (70%). Participants who exhibited a reactive systolic BP response (increase or decrease) tended to have higher baseline systolic BP than participants who exhibited no response (131 mmHg and 138 mmHg versus 124 mmHg, respectively; Table 2). However, only the decrease group had a significantly higher baseline measurement when compared to the no response group (p < 0.001). There was considerable variability in the reactive systolic BP response (range −32– +30 mmHg; Fig. 1). In each pattern of reactive systolic BP response, the reactive response of diastolic BP and pulse pressure followed systolic BP but with a smaller absolute value (Table 2).

**Table 2:** Invasive aortic blood pressure (BP) measurements at baseline, during cuff inflation, and reactive BP response amongst all participants and split into the three groups in response to cuff inflation.

Variable	All (*n* = *234*)	‘No response’ (*n* = *163)*	‘Decrease’ *(n* = *43)*	‘Increase’ (*n* = *28)*	p value (ANOVA)
**Baseline Measurements**					
Systolic BP (mmHg)	127 ± 22	124 ± 20	138 ± 25	131 ± 23	<0.001
Diastolic BP (mmHg)	67 ± 10	66 ± 9	71 ± 11	69 ± 11	0.019
Pulse pressure (mmHg)	59 ± 18	57 ± 17	67 ± 18	61 ± 17	0.005
Heart rate (beats/min)	67 ± 12	67 ± 11	68 ± 12	67 ± 11	0.86
Mean arterial pressure (mmHg)	92 ± 13	90 ± 12	97 ± 15	94 ± 14	<0.001
**Cuff Inflation measurements**					
Systolic BP (mmHg)	127 ± 22	123 ± 20	130 ± 25	139 ± 23	<0.001
Diastolic BP (mmHg)	67 ± 10	66 ± 9	67 ± 12	73 ± 10	0.002
Pulse pressure (mmHg)	59 ± 18	57 ± 17	64 ± 18	66 ± 18	0.011
Heart rate (beats/min)	67 ± 12	67 ± 12	67 ± 13	67 ± 12	0.99
Mean arterial pressure (mmHg)	91 ± 13	89 ± 12	92 ± 16	99 ± 14	<0.001
**Reactive response measurements**					
Systolic BP reactive response (mmHg)	−0.7 ± 6.3	**−**0.3 ± 2.5	−9.2 ± 5.4	9.8 ± 5.1	<0.0001
Diastolic BP reactive response (mmHg)	−0.6 ± 3.5	**−**0.3 ± 2.0	**−**4.9 ± 3.0	4.3 ± 3.3	<0.0001
Pulse pressure reactive response (mmHg)	0.0 ± 3.4	0.0 ± 1.7	−3.2 ± 3.4	4.8 ± 5.1	<0.0001
Heart rate reactive response (beats/min)	−0.2 ± 3.9	0.0 ± 4.2	−1.3 ± 2.6	0.2 ± 3.3	0.19
Mean arterial pressure reactive response (mmHg)	−0.5 ± 4.1	−0.2 ± 2.4	−5.5 ± 3.4	5.5 ± 3.6	<0.0001

Data is expressed as mean ± SD *BP* blood pressure, *ANOVA* analysis of variance. The cuff inflation measurements are the average of the two five-second periods (period 1 and 2) after the start of cuff inflation. The reactive response data were all calculated as (Period 1-Baseline)+(Period 2-Period 1).

### Factors associated with reactive response of systolic BP to cuff inflation

Analysis of BP variability showed significant differences across the three reactive systolic BP response groups whereby at baseline and during cuff inflation the level of systolic and diastolic BP variability was either trending to or significantly higher in the decrease group (Table 3). Analysis of demographic and clinical characteristics across the three reactive systolic BP response groups revealed no significant differences except for a family history of CVD, which was less prevalent in the reactive systolic BP increase group (Table 1). However, after multivariable analysis, no clinical characteristics remained significantly associated with a reactive systolic BP response (Table 4).

**Table 3:** Invasive aortic beat-to-beat blood pressure (BP) variability at baseline, during cuff inflation, and differences amongst all participants and split into the three groups in response to cuff inflation.

Variable	All (*n* = *234*)	‘No response’ (*n* = *163)*	‘Decrease’ *(n* = *43)*	‘Increase’ (*n* = *28)*	p value (ANOVA)
**Baseline Measurements**					
Systolic standard deviation (mmHg)	3.19 (2.1–4.6)	3.02 (2.0–4.1)	4.4 (2.5–6.4)	3.5 (1.9–4.7)	0.005
Diastolic standard deviation (mmHg)	1.56 (1.1–2.2)	1.48 (1.0–2.0)	1.99 (1.5–2.7)	1.55 (1.0–2.3)	<0.001
Systolic coefficient of variation (%)	2.52 (1.8–3.7)	2.41 (1.7–3.4)	3.17 (2.2–4.6)	2.74 (1.5–3.7)	0.09
Diastolic coefficient of variation (%)	2.38 (1.6–3.3)	2.16 (1.5–3.1)	2.97 (2.2–3.7)	2.42 (1.5–3.2)	0.016
**Cuff Inflation measurements**					
Systolic standard deviation (mmHg)	3.08 (2.3–4.9)	2.98 (2.2–4.3)	4.91 (2.8–5.9)	3.44 (2.7–5.2)	<0.001
Diastolic standard deviation (mmHg)	1.59 (1.1–2.2)	1.49 (1.0–2.1)	1.85 (1.3–2.9)	1.93 (1.4–2.3)	0.009
Systolic coefficient of variation (%)	2.63 (1.9–3.9)	2.34 (1.8–3.7)	3.50 (2.3–5.2)	2.65 (2.2–3.3)	0.006
Diastolic coefficient of variation (%)	2.44 (1.7–3.4)	2.30 (1.5–3.1)	3.2 (2.0–4.3)	2.73 (2.1–3.1)	0.014
**Difference in BP variability (cuff inflation – baseline)**					
Systolic standard deviation (mmHg)	0.23 ± 1.7	0.14 ± 1.3	0.42 ± 2.6	0.48 ± 2.4	0.48
Diastolic standard deviation (mmHg)	0.12 ± 1.1	0.09 ± 0.9	0.08 ± 1.5	0.33 ± 0.9	0.54
Systolic coefficient of variation (%)	0.21 ± 1.4	0.14 ± 1.1	0.46 ± 1.8	0.23 ± 1.9	0.40
Diastolic coefficient of variation (%)	0.19 ± 1.5	0.14 ± 1.4	0.32 ± 2.2	0.29 ± 1.2	0.74

Data is exprelssed as median (interquartile range) for non-normally distributed data or mean ± SD where normally distributed.

*BP* blood pressure, *ANOVA* analysis of variance.

**Table 4:** Multivariable regression analysis of the relationship between reactive systolic blood pressure (BP) response (mmHg) and clinical characteristics amongst all study participants.

Variable	β (mmHg)	P value
**Demographic**		
Age (years)	0.0 (−0.1–0.1)	0.44
Sex (male)	1.2 (−0.8–3.3)	0.23
Body mass index (kg/m^2^)	0.0 (−0.2–0.2)	0.82
**Past medical history**		
High BP	1.0 (−1.0–3.1)	0.32
Family history of CVD	−1.5 (−3.4–0.4)	0.12
Type II diabetes mellitus	1.4 (−0.6–3.5)	0.17
Smoking	1.7 (−0.1–3.4)	0.063
Coronary artery disease	−2.0 (−4.0–0.1)	0.059
**Haemodynamic**		
Baseline systolic BP (mmHg)	0.0 (−0.1–0.0)	0.064

### Theoretical classification of high systolic BP

The theoretical misclassification of high systolic BP (≥ 130 mmHg) due to a reactive systolic BP response was observed in 13 participants (5.6%). There were 144 participants with a normal systolic BP at baseline but following cuff inflation eight participants (5.6%) had a high systolic BP. Of these eight participants, four were in the increase group (mean response of +16.5 mmHg) and four were in the no response group (mean response of +3.3 mmHg). On the other hand, of the 90 participants with high systolic BP at baseline, five participants (5.6%) had a normal systolic BP following cuff inflation. Of these five participants, four were in the decrease group (mean response of −12.6 mmHg), and one was in the no response group (mean response of −4.4 mmHg).

### Sensitivity analyses

Use of anti-hypertensive medication was not associated with the magnitude of a reactive systolic BP response (n = 161; p = 0.97). A reactive systolic BP response was not significantly different between participants with or without anti-hypertensive medications, as follows: beta blockers (n = 72; p = 0.56); ACE-inhibitors (n = 49; p = 0.94); angiotensin receptor blockers, (n = 58; p = 0.57); calcium channel blockers (n = 51; p = 0.68); and diuretics, (n = 36; p = 0.80). Forty-five participants had an invasive aortic BP baseline period of <5 s. These participants were evenly distributed among the increase, decrease, and no response groups (Supplemental Table 1). The distribution of the study participants with baseline periods of <5 s vs. ≥5 s was not different between the increase, decrease, and no response groups (p > 0.89). The average response in systolic BP was not different between participants with a short baseline period (−0.3 ± 5.0 mmHg) compared to participants with a longer baseline period (−0.5 ± 5.6 mmHg).

## Discussion

The purpose of this study was to quantify the reactive invasive BP response to cuff inflation because this process may itself cause significant variability in BP measurement. Among 30% of the participants there was a reactive increase (12%) or decrease (18%) in invasive aortic systolic BP upon cuff inflation to an average of approximately 9 mmHg. However, this reactive change in systolic BP had little influence on the theoretical categorization of hypertension status when evaluated with invasive BP. In some individuals the reactive change in systolic BP was substantial and future studies should aim to more fully understand the impact this may have on accurate cuff measured BP.

Studies across several decades have sought to determine if there is a reactive BP response associated with cuff inflation [4–6]. The first study to examine this was performed in 22 hospital in-patients with mild-to-moderate hypertension (mean age 44 years, 77% male) who were left by themselves in a quiet room for the measurement period [6]. Invasive radial BP traces were recorded for a control period of five minutes, then for the minute prior and minute after the start of cuff inflation to show changes through the immediate period of cuff inflation. The inflation of the cuff was automatic for the first two hours of the study and then reprogrammed for a separate two-hour period during which the cuff would only inflate when triggered by the participant. However, there was no reactive response of the invasive radial systolic BP associated with either cuff inflation method. Another study recruited 97 patients receiving postanaesthetic care (mean age 54 years, 55% male) with invasive radial BP recordings [4]. This study automatically recorded 3 cuff measurements each spaced by 5 min before a 30 min rest period and a repeat set of measurements. Throughout each measurement cycle the highest and lowest BPs were recorded and compared to a baseline period recorded prior. The reported mean reactive systolic BP increase (invasive radial) was 6.7 ± 5.9 mmHg. However, 6% of participants also had a reactive decrease in invasive radial systolic BP following cuff inflation, albeit noting that this category of participants included those with a BP decrease of any magnitude (as opposed to at least a 5 mmHg decrease in this current study). Nonetheless, these findings are broadly consistent with the present study, albeit using different methods and reference BP measurement. The decrease group that was observed in the present study accounted for 18% of the study population. Further studies to confirm this observation and to determine the physiological mechanisms behind a decrease in systolic BP caused by cuff inflation are warranted.

The physiological mechanisms that underpin the reactive response associated with cuff inflation remain uncertain. Speculatively, one mechanism that could explain a reactive increase in BP with cuff inflation is increased sympathetic nervous activity (SNA) due to patient discomfort when the cuff is inflated, or even anticipation of cuff inflation. For example, one study showed an increase in systolic BP (measured by finger photoplethysmography) compared to baseline in the 10 s prior to self-measurement of BP, which indicates an anticipatory reactive systolic BP response [12]. However, SNA would be unlikely to explain the reactive systolic BP decrease observed in the present study. This decrease could be related to the drop in heart rate that was observed in this subgroup which may be a sign of peripheral vasodilation and increased vagal activity. A previous study found that hypertensive participants taking beta-blockers, which blocks SNA in the heart, had a non-significantly lower reactive systolic BP increase (measured by finger photoplethysmography) than those receiving other classes of anti-hypertensives [14], but this was not observed in the present study. More generally, participant clinical characteristics were also not different between the reactive response groups. This is contrary to expectations that participants at higher cardiovascular disease risk may display a greater reactive BP response due to exaggerated SNA response to acute stimuli [15, 16]. Additionally, beat-to-beat BP variability was highest among the decrease group, which is not expected if vagal tone is increased. However, BP variability was low in this study which could be due to the relatively short period of data recording or potentially use of mild sedation among nearly all participants. Altogether, the underlying physiological mechanisms and clinical characteristics associated with a reactive systolic BP response to cuff inflation remain unclear and studies that are specifically designed to answer this question, including multiple surrogates of autonomic system activity and other factors such as assessment of anxiety using validated methods, are warranted.

Previous work indicates that baseline systolic BP is not related to the extent of a reactive systolic BP response. Charmoy et al. found that a reactive systolic BP response (defined as the mean of the peak pressure over 10–15 s during cuff inflation) measured by a Finapres device at the finger was greater in individuals with hypertension (either controlled by treatment or uncontrolled with or without treatment) when compared to normotensive individuals. But this reactive systolic BP response was independent of baseline finger systolic BP [14]. Another study showed that baseline invasive radial systolic BP was independent of the magnitude of a reactive BP response [4]. In the present study, participants with a reactive increase or decrease in systolic BP had a non-significantly higher baseline systolic BP than participants in the no reactive systolic BP response group. This finding could potentially be explained by regression to the mean, however, some elements of our data are not consistent with this phenomenon. For the increase group, there was a reactive systolic BP increase of a further 9 mmHg away from the mean. This increase is above the average invasive aortic systolic BP of the study population, and greater than expected if the difference was due to natural BP variability [11]. However, for the decrease group, there was a reactive systolic BP decrease of 9.2 mmHg toward the mean systolic BP of all participants. Future studies that examine multiple cuff inflations and reactive systolic BP responses at different clinical visits could help to confirm if regression to the mean does influence reactive BP responses to cuff inflation.

In the present study, a reactive systolic BP response was observed in 71 (30%) participants, but theoretical misclassification of high systolic BP (≥ 130 mmHg) occurred in only 13 (5.6%) participants. Therefore, the reactive systolic BP response did not have a large impact on the classification of BP. The change in BP in the ‘increase’ and ‘decrease’ groups had, on average, a reactive systolic BP response from 131–139 mmHg. Similarly, participants in the ‘decrease’ group exhibited a reactive response from 138–30 mmHg. This meant that most participants, despite a reactive response of at least 5 mmHg did not cross the 130 mmHg cut point. Nevertheless, the magnitude of reactive change in BP may have relevance. Emerging technologies such as cuffless devices that do not require cuff calibration, if proven to be accurate could help eliminate the changes in BP induced by conventional cuff BP measurement. Cuffless approaches would be of particular interest for nighttime BP measurement where repeated cuff inflations can cause arousal and potentially unreliable recordings in some people.

## Limitations

Study limitations include the use of fluid filled catheters which can introduce errors if not handled correctly. However, the study adhered to expert recommendations, which minimised the risk of measurement errors [9]. An important limitation to consider in this study is the generalisability of the reactive systolic BP response beyond patients undergoing coronary angiography. Indeed, coronary angiography is an invasive procedure with potential clinical and life-altering implications. Therefore, it is expected that many participants in this study may have had anxiety levels above their usual levels, however neither this nor measures of autonomic nervous system activity were quantified in the study. Most participants received mild sedation but the effect of this on our findings is unknown. Conversely, the diagnosis of high BP occurs in a clinic setting and with non-invasive cuff BP. The influence of a reactive systolic BP response on the accuracy of cuff measured BP in a standard setting for hypertension management needs to be determined to make judgements about misclassification of BP in this context. Moreover, reproducibility of the reactive response to cuff inflation should be assessed in future studies, ideally at separate clinical visits, which was not feasible in this study.

The present study provides new insights into the reactive invasive aortic BP response to cuff inflation. While the overall impact on hypertension classification was minimal, the presence of a reactive BP response in 30% of participants highlights its potential significance. Given the clinical relevance of even minor changes in systolic BP, future, prospectively designed studies should focus on elucidating the factors contributing to these reactive responses and their implications for hypertension management.

## Summary

### What is known about the topic

- Few studies have examined blood pressure cuff inflation itself as a potential source of measurement inaccuracy.
- Only one study used intra-arterial aortic blood pressure measurements as the reference standard, but the cuff inflation/deflation cycle was not performed as recommended for standard cuff blood pressure measurement.

### What this study adds

- The present study provides new insights into the reactive invasive aortic blood pressure response to cuff inflation.
- Although the blood pressure response to cuff inflation had minimal impact on hypertension classification, a reactive response was observed in 30% of participants, highlighting its potential clinical relevance.
- Given the clinical importance of even small changes in systolic blood pressure, future research should identify factors contributing to these reactive responses and their importance for hypertension management.

## Supplementary information

Supplemental material

## Data Availability

The datasets generated during and/or analysed during the current study are available from the corresponding author on reasonable request.
