# Supplementary material for: Invasive observation of reactive systolic blood pressure responses to upper-arm cuff inflation
Source: J Hum Hypertens. 2026 Jun 17;40(8):663–9. doi: 10.1038/s41371-026-01172-3 (PMC13457076; doi:10.1038/s41371-026-01172-3)
Supplement: Supplementary file 1 [file 41371_2026_1172_MOESM1_ESM.docx]

**Supplemental material**

**Invasive observation of reactive systolic blood pressure responses to upper-arm cuff inflation**

Louisa F. Morris,^1*^ Matthew K. Armstrong,^2*^ James E. Sharman,^1^ Martin G. Schultz,^1,3^ J. Andrew Black,^1,4^ Nathan B. Dwyer,^1,4^ Philip Roberts-Thomson,^4^ Heath Adams,^1,4^ Stéphane G. Carlier,^5^ Dean S. Picone^6,7^

*These authors contributed equally to this work

**Institutional affiliations**

^1^ Menzies Institute for Medical Research, University of Tasmania, Hobart, Australia.

^2^ Department of Exercise Science, Falk College of Sport, Syracuse University, Syracuse, USA

^3^ Department of Rural Health, University of Melbourne, Shepparton, Australia.

^4^ Royal Hobart Hospital, Hobart, Australia.

^5^University of Mons (UMons), Faculty of Medicine, Department of Cardiology, Mons, Belgium .

^6^ School of Health Sciences, Faculty of Medicine and Health, The University of Sydney, Sydney, Australia

^7^ Westmead Applied Research Centre, The University of Sydney, Sydney, Australia

**Table of contents**

**Supplemental Table 1.** Reactive BP response compared between participants with a baseline BP measurement period of <5 seconds vs. participants with a baseline BP measurement period of >5 seconds

| **Supplemental Table 1.** Reactive BP response compared between participants with a baseline BP measurement period of <5 seconds vs. participants with a baseline BP measurement period of >5 seconds. | | | |
| --- | --- | --- | --- |
| **Variable** | **Baseline period <5 seconds** | **Baseline period >5 seconds** | **p value (ANOVA)** |
| **SBP reactive response (mmHg)** | -0.3 ± 5.0 | -0.3 ± 5.6 | 0.83 |
| **DBP reactive response (mmHg)** | -0.2 ± 2.7 | -0.4 ± 3.0 | 0.62 |
| **PP reactive response (mmHg)** | -0.1 ± 3.3 | 0.0 ± 3.5 | 0.94 |
| **HR reactive response (bpm)** | -0.2 ± 6.4 | -0.2 ± 3.1 | 0.97 |
| **MAP reactive response (mmHg)** | 0.0 ± 4.2 | -0.7 ± 4.0 | 0.30 |
| Data is expressed as mean ± SD. SBP = systolic blood pressure; DBP = diastolic blood pressure; BPV = blood pressure variability; PP = pulse pressure; HR = heart rate; MAP = mean arterial pressure; ANOVA = analysis of variance. Significant difference between the two groups was considered to be p>0.05. | | | |
